# Preliminary validation of the Dutch version of the Posttraumatic stress disorder checklist for DSM-5 (PCL-5) after traumatic brain injury in a civilian population

**DOI:** 10.1371/journal.pone.0231857

**Published:** 2020-04-20

**Authors:** Dominique L. G. Van Praag, Haghish Ebad Fardzadeh, Amra Covic, Andrew I. R. Maas, Nicole von Steinbüchel

**Affiliations:** 1 Department of Neurosurgery, Antwerp University Hospital and University of Antwerp, Edegem, Belgium; 2 Institute of Medical Psychology and Medical Sociology, University Medical Center Göttingen (UMG)/Georg-August-University, Göttingen, Germany; Monash University, AUSTRALIA

## Abstract

The Posttraumatic stress disorder checklist (PCL) is the most widely used questionnaire to screen for symptoms of posttraumatic stress disorder (PTSD), based on the Diagnostic and Statistical manual of Mental disorders (DSM-IV) criteria. In the latest edition of the DSM (DSM-5), the criteria for PTSD were revised leading to the development of the PCL-5. So far, there is no validated Dutch version of the PCL-5. The aim of this study is to determine psychometric characteristics of the Dutch translation and linguistic validation of the PCL-5 and to evaluate internal consistency, criterion and structural validity. In a population of 495 civilian, traumatic brain injury patients, the PCL-5, the Generalized anxiety disorder questionnaire and the Depression scale of the Patient health questionnaire were administered. The PCL-5 was translated in Dutch following a strict procedure of linguistic validation and cognitive debriefing. Results show an excellent internal consistency and high criterion validity. Confirmatory factor analysis demonstrated a good fit for the four-factor DSM-5 model, but a superior fit for the six-factor Anhedonia model and the seven-factor Hybrid model, similar to the English version of the PCL-5. Preliminary validation of the Dutch translation of the PCL-5 was proven to be psychometrically sound and can be used for clinical and academic purposes, specifically for TBI patients. Future research should examine concurrent and discriminant validity for the Dutch translation in broader populations at risk for PTSD, and include a structured interview to evaluate diagnostic utility.

## Introduction

Posttraumatic Stress Disorder (PTSD) is a mental disorder following a traumatic event, expressed by different symptoms such as persistent remembering or reliving the event, trying to avoid specific stressors, difficulty in falling asleep, irritability, hyper-vigilance and others [1]. The topic of PTSD has been widely investigated in patients following a traumatic brain injury (TBI) [2]. TBI forms a large public health and societal problem and over two million people are admitted to hospital each year in Europe [3]. Reported prevalence rates of PTSD following civilian TBI range from 0 to 50% [4–11]. There is no clear evidence for effects of the severity of TBI on the development of PTSD. For mild TBI, prevalence rates range from 4 to 34% [12–28], and for moderate to severe TBI from 0 to 25% [13,25,26,29–39]. In military settings, the incidence rate is higher and ranges from 33 to 65% [40,41]. Despite broad ranges of reported incidence rates of PTSD after TBI, PTSD is recognized as an important cause of disability following TBI [42].

The Posttraumatic stress disorder checklist (PCL) is the most widely used questionnaire to screen for symptoms of PTSD in research and clinical contexts [43]. The original 17-item version of the PCL questionnaire was based on the Diagnostic and Statistical manual of Mental disorders–fourth edition (DSM-IV) criteria [44]. The symptoms can be categorized into three DSM-clusters: Intrusive recollections, Avoidance/Numbing and Arousal. The majority of structural validity studies however, support a four factor model [45,46].

In the fifth edition of the DSM, diagnostic criteria for PTSD were revised, and a new category of Trauma- and Stressor-Related disorders was integrated [1]. PTSD can be diagnosed following exposure to a traumatic event, e.g. (threatened) death, or (threatened) serious injury, and now includes four factors (Intrusion, Avoidance, Negative alterations in cognitions and mood, and Arousal) [45,46]. The most prominent change concerns anhedonic/dysphoric symptoms which are reflected by negative cognitions and mood states. Examples of negative appraisals are ‘nobody can be trusted’ or ‘nothing good can happen to me’. Negative emotions can occur and include anger, shame, guilt, or the inability to experience positive emotions. Symptoms need to be present for more than one month, need to cause significant symptom-related distress or functional impairment, and cannot be initiated by medication, substance or illness. In accordance to the DSM-5, the PCL underwent several changes and is now composed of 20 items (PCL-5) [47].

The PCL-5 was developed and published in English and has been translated in French [48], in German [49], in Swedish [50], in Chinese [51,52], in Brazilian [53], in Turkish [54], in Arab and Kurdish [55] and in Filipino [56]. However, these translations have not been validated in TBI. Up till now, there was no validated Dutch version of the PCL-5. This may be considered highly relevant as it confounds comparability of Dutch studies to those from other countries, and poses challenges to clinicians wishing to screen Dutch speaking patients after TBI. The objective of the present study is to determine psychometric characteristics of the Dutch translation of the PCL-5 and to evaluate the quality of the translation, the linguistic validation and the psychometrics. Internal consistency, criterion validity and structural validity will be investigated.

## Materials and methods

### Participants

Data for the current study were collected as sub-study within the European CENTER-TBI Core study (Collaborative European NeuroTrauma Effectiveness Research: www.center-tbi.eu), an observational prospective study that aims to better characterize TBI and identify the most effective clinical interventions for managing TBI (clinicaltrials.gov NCT02210221) [57]. The CENTER-TBI Core study collected data on 4509 patients from 65 hospitals across 19 countries over a 3 year period from dec 2014 to dec 2017. All patients with a clinical diagnosis of TBI, an indication for a CT-scan and presentation to the study centre within 24 hours of the injury were eligible for enrollment. Pre-existing severe neurological disorders were an exclusion criterion. Informed consent was obtained in all subjects according to local and national requirements.

On enrollment, patients were stratified by care pathway into three strata. The ER stratum included patients that visited the emergency department and were discharged the same day. The admission stratum included patients that were admitted to the ward and the ICU stratum included patients primarily admitted to the intensive care unit.

For the current study, we selected adult patients (≥ 16 years of age) from the Dutch speaking centers. The analyzed data came from seven clinics in the Netherlands and two clinics in Dutch speaking cities in Belgium (Antwerp University Hospital and University Hospital Leuven).

### Procedure

The CENTER-TBI study aimed to follow patients up to two years post injury. At different time points, self-report questionnaires and neuropsychological assessments were performed depending on the stratum of inclusion. All included patients were asked to complete the PCL-5, the Patient health questionnaire-9 (PHQ-9) [58] and the Generalized anxiety disorder questionnaire-7 (GAD-7) [59] at six months post injury.

The medical assessments of the study were performed by physicians and other assessment by research personnel, who were trained to collect and accurately enter data in the electronic case report form.

Participants were invited to revisit the hospital for an interview at the scheduled follow up time points and were examined by a research nurse or a neuropsychologist. Research nurses or neuropsychologists collecting outcome data were extensively trained in collecting psychological data and administering cognitive tests. If a visit was not possible or the patient refused to perform the neuropsychological assessments, participants received the PCL-5 instrument among other questionnaires by mail with a pre-stamped return envelope. In case the patient did not return the questionnaire(s) or did not respond to all questions, they were contacted to obtain the missing data. All data entered in the electronic case report form were de-identified and stored in a secure database.

### Ethical approval

The CENTER-TBI study (EC grant 602150) has been conducted in accordance with all relevant laws of the EU if directly applicable or of direct effect and all relevant laws of the country where the recruiting sites were located, including but not limited to, the relevant privacy and data protection laws and regulations (the “Privacy Law”), the relevant laws and regulations on the use of human materials, and all relevant guidance relating to clinical studies from time to time in force including, but not limited to, the ICH Harmonized Tripartite Guideline for Good Clinical Practice (CPMP/ICH/135/95) (“ICH GCP”) and the World Medical Association Declaration of Helsinki entitles “Ethical Principles for Medical Research Involving Human Subjects”. Informed Consent was obtained for all patients recruited in the Core Dataset of CENTER-TBI and documented in the electronic case report form. Ethical approval was obtained for each recruiting sites. The list of sites, Ethical Committees, approval numbers and approval dates can be found on the website: https://www.center-tbi.eu/project/ethical-approval.

### Measures

Self-report questionnaires administered included the PCL-5 instrument, the PHQ-9, GAD-7, and a socio-demographic questionnaire.

#### PTSD checklist for DSM-5 (PCL-5)

The PTSD Checklist for DSM-5 (PCL-5) is a self-report measure to screen for PTSD, to determine symptom severity of PTSD, to monitor symptom change after treatment or to make a provisional PTSD diagnosis [47]. The PCL-5 includes 20 items that reflect the DSM-5 diagnostic criteria of PTSD. Patients are asked how much they have been bothered by each item over the past month. Items are scored on a Likert scale ranging from 0 to 4, where higher scores indicate more pronounced PTSD symptoms. The sum of scores can range from 0 to 80. A cut-off score of 31 was suggested to best screen for PTSD with a specificity of .95, a sensitivity of .85 and an efficiency of .95 [48]. Related to the DSM-5, the PCL-5 can also be interpreted using the Symptom Cluster Method to screen for PTSD and provide a provisional diagnosis. An item with a score of 2 or higher is considered clinically relevant. A diagnosis of PTSD requires at least one intrusion item, at least one avoidance item, two or more negative alterations in cognitions and mood, and two or more arousal symptoms. A formal diagnosis however, needs a more thorough evaluation for example by using the Clinician-Administered PTSD Scale (CAPS) [49].

#### Patient health questionnaire (PHQ-9)

The Patient health questionnaire (PHQ-9) is a self-report questionnaire to screen for depression [58]. The PHQ-9 consists of nine items, related to the DSM-IV criteria, asking how often the patient was bothered by the symptom. Items can be scored from 0 ‘Not at all’ to 3 ‘Almost each day’, resulting in a total score of 0 to 27. A high score suggests a depressive disorder. The PHQ-9 shows a good agreement with assessments by mental health professionals (K = .65) [60]. Internal consistency was high and produced Cronbach alphas from .86 to .89 [58]. The Dutch translation of the PHQ-9 has previously been validated, showing similar internal consistency (C’s α = 0.83) and interrater reliability (K = 0.81), as the English version [61,62].

#### Generalized anxiety disorder questionnaire (GAD-7)

The Generalized anxiety disorder questionnaire (GAD-7) is a self-administered patient questionnaire, used as a screening tool and severity measure for anxiety [59]. Patients are asked whether they are bothered by one of the seven items, related to DSM-IV Generalized anxiety disorder. The items are scored on a scale from 0 meaning ‘Not at all’ to 3 indicating ‘Almost each day’. A high total score refers to a possible anxiety disorder with a maximum score of 21. Internal consistency is excellent (α = .89) and confirmatory factor analysis produced a one-dimensional structure of the GAD-7 [63]. A cut-off score of 7 or higher yielded a sensitivity of .73 and a specificity of .67 [64]. The Dutch version of the GAD-7 has shown good reliability (C’s α = 0.86) and good convergent validity (r = 0.82) [65,66].

#### Sociodemographic questionnaire

Patients’ socio-demographic data including gender, age, race, marital and family status as well as patients’ level of education and parents’ level of education was gathered. Furthermore, patients were asked about pre-injury employment status, previous history of psychiatric disorders, and also the cause of injury.

### Translation and linguistic validation

To ensure the linguistic validity of the translated instrument, a strict procedure was specified prior to the translation of PCL-5 from English to Dutch, including linguistic validation and cognitive debriefing. First, two independent forward translations of PCL-5 were created by native Dutch speakers in the medical academic field. Reviewing the translations, the consensus version was developed by combining the translations. Furthermore, the consensus version was reviewed and edited by a psychologist collaborating face-to-face with a translator to ensure the conceptual equivalence of the translated version. Next, the consensus version was back-translated to English by a native English speaker and after comparing it to the source instrument, it was approved by the linguistic validation team at UMG.

To carry out the cognitive debriefing, three healthy volunteers and three patients with PTSD were examined using the translated questionnaire and clinical interview. Further adjustments were made by two professional translators after reviewing the results of the cognitive debriefing, which were approved by an expert panel. Final harmonization was applied by five language coordinators of the UMG linguistic validation team at the UMG and finally approved by the study board.

### Statistical analysis

Sample characteristics were described for gender, age, level of education, injury type, racial background and prior mental health status. In order to evaluate the validity and reliability of the PCL-5 translation, its properties were examined and reported both at item-level and scale level.

#### Reliability

For the item-level analysis, items distribution and skewness are reported. For scale-level analyses, internal consistency of the scale was examined using Cronbach’s alpha and split-half reliability. Furthermore, item-total correlations and Cronbach’s alpha—if item omitted–is reported.

#### Criterion validity

We examined the criterion validity of the PCL-5 instrument by reporting its correlations with two related instruments, GAD-7 and PHQ-9. Previous research has reported high correlations ranging from 0.70 to 0.77 between PTSD and depression symptoms using PCL-5 and PHQ-9 instruments [56,67,68]. Similar correlations ranging from 0.61 to 0.67 have been reported between PTSD and anxiety symptoms by using PCL-5 and GAD-7 instruments [56,67,68].

#### Structural validity

The structural validity of the PCL-5 instrument was analyzed using confirmatory factor analysis (CFA). We applied the DSM-5 four-factor model, the six-factor Anhedonia model [54], and the seven-factor Hybrid model [69]. The Anhedonia model includes Intrusion, Avoidance, Negative affect, Anhedonia, Dysphoric arousal, and Anxious arousal factors. The seven-factor hybrid model is very similar to the anhedonia model and differs in only two items. Namely, it suggests an additional factor of Externalizing behavior by extracting two items from the Dysphoric arousal in the six-factor model.

In all of the CFA models, items were specified to a single factor only and the latent variables were specified to correlate with one another. A number of criteria were considered for evaluating the fitness of the factor analyses. In addition to Chi-Square goodness of fit, the Root Mean Square Error of Approximation (RMSEA) and its confidence interval as well as Standardized Root Mean Square Residual (SRMR), Comparative Fit Index (CFI) and Tucker Lewis Index (TLI) were investigated. The CFA analyses were carried out using R version 3.4.1 and the lavaan package [70] version 0.5.23 with WLSMV estimator, because the data was ordinal. As a result, the Akaike information criterion (AIC) and Bayesian information criterion (BIC) that are only available for maximum likelihood estimator were not computed. Based on the recommended cut-off suggested in the literature, the CFA model fit was considered adequate for CFI and TLI values above 0.95, RMSEA value less than 0.06, and SRMR value below 0.08 [71].

## Results

### Sample characteristics

Of the 815 TBI patients admitted to the Dutch-speaking hospitals, 320 subjects (99 females and 204 males) did not complete the questionnaires at the 6-months follow up after the injury and thus were dropped from the analysis. The analysis included 495 subjects, where 419 subjects were included in the Netherlands and 76 subjects in Antwerp and Leuven, two Dutch speaking cities in Belgium. From the participants, 186 were female (median age = 58, IQR = 31) and 309 were male (median age = 50, IQR = 37). The sample characteristics are summarized in Table 1.

**Table 1:** Characteristics of the study population.

Characteristics		Number	Percentage
	Male	309	62.4
	Female	186	37.6
	Missings	0	0
Age	16–30	93	18.8
	31–44	54	10.9
	> 45	335	67.7
	Missings	0	0
Level of education	Up to high school	140	28.3
	Technical training	149	30.1
	College / University	150	30.3
	Missings	49	9.9
Hospital admission type	Emergency	81	16.4
	Admission	249	50.3
	ICU	165	33.3
	Missings	0	0
Injury type	Closed	482	97.4
	Closed with open depressed skull fracture	2	0.4
	Penetrating	2	0.4
	Penetrating-perforating	1	0.2
	Penetrating-tangential	0	0
	Blast	0	0
	Crush	4	0.8
	Missings	2	0.4
Racial background	Asian	12	2.4
	Black	13	2.6
	Caucasian	461	93.1
	Missings	7	1.4
Prior mental health problems	Yes	118	23.8
	No	346	69.9
	Missings	31	6.3

As shown in Table 1, the majority of the subjects were caucasian and over 45 years of age, and 97.4% of the subjects had a closed head injury.

While the PCL-5 score can range from 0 to 80, the score ranged from 0 to 72 in our study population. More importantly, the majority of the subjects scored low on the PCL-5 scale (median = 7, mean = 11.25, SD = 13.06). Therefore, the distribution of the PCL-5 score was right-skewed (skew = 1.82). Only 45 patients (9%) had a total score of 31 or higher, suggested as best cut-off score to screen for PTSD [48]. As expected, the PCL-5 score also varied based on the demographics of the study population. For example, the PCL-5 score was found to be negatively correlated with age (r = -0.20) and level of education (r = -0.12) and positively correlated with prior history of mental health problems (r = 0.21). However, the correlation of the PCL-5 score with gender was near 0.

### Reliability

The translated scale shows an excellent internal consistency of 0.93. Table 2 summarizes the Cronbach's Alpha for the total score and each of the subscales of the PCL-5 instrument. As shown below, all of the subscales have a good internal consistency. In addition, using split-half method on all of the items reveals a high reliability of 0.96 for the total scale.

**Table 2:** Internal consistency of the PCL-5 instrument.

PCL—5 subscales	Alpha
**Intrusive**	0.90
**Avoidance**	0.80
**Cogn/Mood**	0.84
**Arousal**	0.79
**Total**	0.93

Further details about the scale reliability are provided in Table 3. The table shows the mean, SD, item-total correlation, Cronbach's alpha of the scale if each item is dropped, as well as Skewness and Kurtosis of each translated PCL-5 item. The low mean and the positive skewness values of each item are in-line with the total score of the scale, indicating that the TBI patients scored low on PCL-5 instrument. Nevertheless, a high positive item-total correlation and a constant Cronbach's alpha of 0.93 provide further information about the scale's reliability.

**Table 3:** Item descriptive statistics.

	Mean	SD	Item-total correlation	Alpha if item omitted	Skewness	Kurtosis
**1 Repeated memories**	0.66	1.01	0.73	0.93	1.71	2.37
**2 Repeated dreams**	0.30	0.76	0.69	0.93	2.92	8.58
**3 Reliving experience**	0.38	0.79	0.72	0.93	2.46	6.05
**4 Upset when reminded**	0.36	0.80	0.8	0.93	2.52	6.10
**5 Physical reaction when reminded**	0.30	0.74	0.74	0.93	2.96	8.86
**6 Avoiding memories**	0.46	0.90	0.69	0.93	2.24	4.65
**7 Avoiding external reminders**	0.45	0.95	0.71	0.93	2.27	4.41
**8 Trouble remembering**	0.85	1.30	0.56	0.94	1.43	0.69
**9 Negative beliefs**	0.39	0.86	0.70	0.93	2.51	6
**10 Blaming yourself**	0.44	0.93	0.55	0.93	2.30	4.53
**11 Negative feelings**	0.46	0.93	0.77	0.93	2.25	4.52
**12 Loss of interest**	0.73	1.12	0.72	0.93	1.52	1.31
**13 Feeling distant**	0.49	0.94	0.68	0.93	2.16	4.12
**14 Trouble positive feelings**	0.46	0.93	0.72	0.93	2.31	4.88
**15 Irritable behavior**	0.58	0.97	0.68	0.93	1.81	2.56
**16 Risk taking**	0.19	0.56	0.51	0.93	3.67	15.16
**17 Being superalert**	0.99	1.22	0.61	0.93	1.12	0.22
**18 Feeling jumpy**	0.62	0.99	0.75	0.93	1.71	2.39
**19 Difficulty concentrating**	1.13	1.22	0.70	0.93	0.94	-0.14
**20 Trouble sleeping**	1.04	1.29	0.62	0.93	1.01	-0.25

### Criterion validity

For the GAD-7 and PHQ-9 instruments, the total score ranged from 0 to 21 (maximum possible score of 24) and 27 (maximum possible score of 27) respectively. However, the majority of the subjects scored low on these scales and the median score was 2 for GAD-7 and 3 for PHQ-9.

Both male (median = 6, IQR = 15) and female (median = 8, IQR = 13) participants had similar PTSD symptom scores, the median scores for the total PCL-5 score (Mann-Whitney U-test: *χ*^2^ = 1.473, p = 0.225) and subscales intrusion, avoidance, negative alterations in cognition/mood, arousal (resp. *χ*^2^ = 2.027, p = 0.154, *χ*^2^ = 0.720, p = 0.396, *χ*^2^ = 0.186, p = 0.666, *χ*^2^ = 0.781, p = 0.377) did not differ significantly for males and females. However, females mean score was significantly higher for GAD-7 (*χ*^2^ = 8.637, p = 0.003) and for the PHQ-9 (*χ*^2^ = 5.115, p = 0.024).

Based on the previous findings, high correlations were expected between scores of PTSD and depression [48,50] and PTSD and anxiety symptoms [54]. Table 4 shows the correlation matrix between these variables, which were all statistically significant.

**Table 4:** Correlations between the instruments.

	PCL-5	GAD-7	PHQ-9
**PCL-5**	1	0.71	0.72
**GAD-7**	0.71	1	0.80
**PHQ-9**	0.72	0.80	1

### Structural validity

Multiple confirmatory factor analyses were applied to examine the factor structure of the PCL-5 questionnaire. The factor analysis for the four-factor DSM-5 model, reveals acceptable CFI, SRMR and RMSEA, showing an adequate fit for the model. However, the Chi-Square statistics indicate that the model fit is not ideal (*χ*^2^ = 246.49, p<0.000) [72]. The six-factor Anhedonia model (*χ*^2^ = 152.97, p< 0.531) and the seven-factor Hybrid model (*χ*^2^ = 138.631, p<0.718), both show a superior goodness-of-fit for the model compared to the four-factor DSM-5 model (Table 5). The Scaled Chi-Square Difference Test revealed that with p<0.001, the six-factor Ahnhedonia and the seven-factor Hybrid models fit significantly better than the four-factor DSM-5 and six-factor Anhedonia models respectively.

**Table 5:** Results of CFA for four-, six-, and seven-factor PTSD models.

Models	*χ*^*2*^^*a*^ (df)	RMSEA^b^	RMSEA 95% CI^c^	CFI^d^	TLI^e^	SRMR^f^	P
**Four-factor DSM-5 model**	246.49 (164)	0.032	0.023–0.040	0.998	0.998	0.050	0.000
**Six-factor Anhedonia model**	152.97 (155)	0.000	0.000–0.020	1.000	1.000	0.041	0.531
**Seven-factor Hybrid model**	138.63 (149)	0.000	0.000–0.017	1.000	1.000	0.039	0.718

^a^chi-square statistics

^b^Root Mean Square Error of Approximation

^c^Confidence interval

^d^Comparative fit index

^e^Tucker Lewis index

^f^Standardized Root Mean Square Residual

Table 6 shows the standardized estimates of the models. The correlations between the latent variables within each model were very high and range from 0.77 to 0.94 for the four-factor DSM-5 model (S1 Table), from 0.70 to 0.93 for the six-factor Anhedonia model (S2 Table), and from 0.70 to 0.98 for the seven-factor Hybrid model (S3 Table).

**Table 6:** Estimated factor loadings of each item for four, six, and seven-factor PTSD models.

	Four-factor DSM-5 model	Six-factor Anhedonia model	Seven-factor Hybrid model
**1 Repeated memories**	0.8607	0.8606	0.8605
**2 Repeated dreams**	0.8731	0.8733	0.8734
**3 Reliving experience**	0.8661	0.8669	0.8669
**4 Upset when reminded**	0.9481	0.9474	0.9475
**5 Physical reaction when reminded**	0.9048	0.9050	0.9050
**6 Avoiding memories**	0.8795	0.8797	0.8797
**7 Avoiding external reminders**	0.8943	0.8941	0.8941
**8 Trouble remembering**	0.6388	0.6453	0.6456
**9 Negative beliefs**	0.8355	0.8447	0.8448
**10 Blaming yourself**	0.6892	0.6930	0.6930
**11 Negative feelings**	0.8931	0.9019	0.9016
**12 Loss of interest**	0.8173	0.8603	0.8607
**13 Feeling distant**	0.8200	0.8609	0.8611
**14 Trouble positive feelings**	0.8471	0.8955	0.8948
**15 Irritable behavior**	0.7664	0.7775	0.8232
**16 Risk taking**	0.6684	0.6783	0.7362
**17 Being superalert**	0.6706	0.7875	0.7693
**18 Feeling jumpy**	0.8252	0.7076	0.6720
**19 Difficulty concentrating**	0.7749	0.7186	0.7186
**20 Trouble sleeping**	0.6964	0.8941	0.8942

## Discussion

### Properties of the PCL-5

In this study, we performed a preliminary validation of the Dutch translation of the PCL-5 instrument for a civilian sample of 495 patients with TBI from the Netherlands and Belgium.The Dutch translation was proven to be psychometrically sound as it demonstrated excellent internal consistency and reliability and high criterion validity. This has important implications for future, international research on PTSD in a TBI population, as this version is now available in Dutch. Confirmatory Factor Analysis showed a good fit for the most frequently tested models; four-factor DSM-5 model, the six-factor Anhedonia model and the seven-factor Hybrid model. The latter are considered to be a superior fit compared to the DSM-5 model. The results are similar to those of the original, English version [48,73].

Overall, the patients reported low anxiety, low depression, and low PTSD symptoms causing the score distribution to be positively skewed. However, these scores were very close to the predicted correlations, providing support for the criterion validity of the instrument. The correlation of PCL-5 with anxiety and depression was found to be 0.71 and 0.72, respectively. These correlations are similar to those reported by Ashbaugh and colleagues (2016) using the English and French version of the PCL-5 and the Impact of Event Scale–Revised and the Center for Epidemiological Studies—Depression Scale, supporting convergent and divergent validity (S4 Table) [48,74,75]. Hall and collegeagues translated the PCL-5 to Filipino and used the same questionnaires to determine criterion validity; PHQ-9 and GAD-7 and found similar correlations (resp. 0.71 and 0.61) [56]. The correlations between the Turkish version of the PCL-5 and the Beck Depression Inventory [76] and Beck Anxiety Inventory [54,77], and the Arab/Kurdish version of the PCL-5 and the Depression Hopkins Symptom checklist [55,78], and the Swedish version of the PCL-5 and the Montgomery-Asberg Depression Rating Scale [50,79] range from 0.60 to 0.81. Nine percent of the patients showed a total score of 31 or higher which suggests a possible diagnosis of PSTD. This finding is in line with reported prevalence rates of studies measuring PTSD using the PCL in civilian TBI populations (0–22.7%) [17,22,25,26,29,32,80–82].

The PCL-5 was not associated with female gender as was found in previous studies [83]. Previous findings suggesting older age is a risk factor to develop PTSD is not supported, older patients show a slightly lower total score for PTSD symptoms [84]. Patients with a lower education level are more likely to develop PTSD symptoms. A history of mental health problems was associated with higher PTSD symptoms, and confirms previous findings [30,84].

Moreover, the internal consistency of the Dutch version of the PCL-5 is within the range of the internal consistency of the English instrument (α = 0.93–0.95) and other versions of the PCL-5 [48,73]. Psychometric evaluations of the French, German, Swedish, Filipino and Turkish translations show excellent internal consistency for the total score (α = 0.90–0.95). The Cronbach’s alpha of the subscales of the PCL-5 were very similar to those reported for the French (α = 0.79 to 0.87), the English (α = 0.81 to 0.90), the German (α = 0.79 to 0.89) and the Turkish version (α = 0.78 to 0.87) ranging from 0.79 to 0.90 [48–50,54].

The current study also confirms the structural validity of the PCL-5 in a Dutch, civilian TBI sample. The results of the CFA analysis show that the four-factor DSM-5 PTSD model as well as the six-factor Anhedonia and seven-factor Hybrid models, all provide an adequate fit. Apart from Chi-Square, which was significant for the DSM-5 model, all the indexes were within the defined cut-offs for the defined adequate fit. Moreover, the analysis also confirms previous findings of the French and English version [48], and the Chinese version [51,52] that the six-factor Anhedonia model and the seven-factor Hybrid model both are superior to the four-factor DSM-5 model [48].

### Limitations

The current manuscript made use of data of a larger study and the data was not specifically collected for validating the new instrument. As a result, some of the routine validation and reliability procedures such as convergent and divergent validity were not performed, since they require additional resources, i.e. including more questionnaires. Test-retest reliability test was not performed, since only four patients completed the retest PCL-5 ten days after the first PCL-5. The Dutch version of the PCL-5 was not validated against a structured interview for PTSD to evaluate diagnostic utility. The PCL-5 is a checklist and therefore should not be used as diagnostic tool. However, the reported reliability measures, the magnitudes of the correlations and the factor loadings, as well as the results of the confirmatory factor analysis, all provide evidence for the reliability and the validity of the instrument for the Dutch translation of the PCL-5 instrument.

There was a relatively high number of patients who did not return the questionnaires or attend the follow-up visit. However, compared to other observational studies, this is a reasonable response rate in health research [85].

Research in patients with TBI implies a certain risk for spurious results because of cognitive difficulties. Concentration problems, impulsivity or the tendency to underestimate their functional problems may influence the PCL-5 total score [6].

### Conclusions and outlook

The psychometric properties of the Dutch translation of the PCL-5 show solid reliability and criterion validity in a population of civilian TBI patients. The PCL-5 now can be used for clinical purposes, particularly in Dutch speaking TBI patients, to quantify PTSD symptom severity and to screen for PTSD.

Future research should complete psychometric evaluation by examining concurrent and discriminant validity for the Dutch translation in broader populations at risk for PTSD, and should include a structured interview as a measure for PTSD to evaluate diagnostic utility. Validating against a diagnostic tool for PTSD will allow determination of the best cut-off score for the Dutch version of the PCL-5 in a civilian TBI population. In addition, longitudinal research should evaluate the sensitivity to change over time. Validating the PCL-5 in broader populations is relevant to increase generalizability. A fully validated PTSD instrument will allow researchers to reliably estimate the prevalence of PTSD, compare Dutch samples to those from other regions, and facilitate international collaborative studies.

## Supporting information

S1 Table Latent variable correlations in four-factor DSM-5 model. (PDF)

S2 Table Latent variable correlations in six-factor Anhedonia model. (PDF)

S3 Table Latent variable correlations in seven-factor Hybrid model. (PDF)

S4 Table Correlations of depression and anxiety instruments with PCL-5. (PDF)

S1 File Dutch translation of the PCL-5. (PDF)

S2 File (PDF)
