# Supplementary material for: Preliminary validation of the Dutch version of the Posttraumatic stress disorder checklist for DSM-5 (PCL-5) after traumatic brain injury in a civilian population
Source: PLoS One. 2020 Apr 20;15(4):e0231857. doi: 10.1371/journal.pone.0231857 (PMC7170250; doi:10.1371/journal.pone.0231857)
Supplement: S1 Table — (PDF) [file pone.0231857.s001.pdf]

S1 Table. Latent variable correlations in four-factor DSM-5 model

	Intrusive	Avoidance	CogMood	Arousal
**Intrusive**	1.000			
**Avoidance**	0.902	1.000		
**CogMood**	0.816	0.768	1.000	
**Arousal**	0.845	0.790	0.940	1.000
