# Supplementary material for: Preliminary validation of the Dutch version of the Posttraumatic stress disorder checklist for DSM-5 (PCL-5) after traumatic brain injury in a civilian population
Source: PLoS One. 2020 Apr 20;15(4):e0231857. doi: 10.1371/journal.pone.0231857 (PMC7170250; doi:10.1371/journal.pone.0231857)
Supplement: S2 Table — (PDF) [file pone.0231857.s002.pdf]

S2 Table. Latent variable correlations in six-factor Anhedonia model

	Intrusive	Avoidance	NegAffect	Anhedonia	DysArous1	AnxArous
**Intrusive**	1.000					
**Avoidance**	0.902	1.000				
**NegAffect**	0.853	0.789	1.000			
**Anhedonia**	0.716	0.696	0.869	1.000		
**DysArous1**	0.810	0.738	0.919	0.926	1.000	
**AnxArous**	0.812	0.783	0.823	0.779	0.865	1.000
