# Supplementary material for: Preliminary validation of the Dutch version of the Posttraumatic stress disorder checklist for DSM-5 (PCL-5) after traumatic brain injury in a civilian population
Source: PLoS One. 2020 Apr 20;15(4):e0231857. doi: 10.1371/journal.pone.0231857 (PMC7170250; doi:10.1371/journal.pone.0231857)
Supplement: S3 Table — (PDF) [file pone.0231857.s003.pdf]

S3 Table. Latent variable correlations in seven-factor Hybrid model

	Intrusive	Avoidance	NegAffect	Anhedonia	DysArous2	ExtBehav	AnxArous
**Intrusive**	1.000						
**Avoidance**	0.902	1.000					
**NegAffect**	0.853	0.789	1.000				
**Anhedonia**	0.716	0.696	0.869	1.000			
**DysArous2**	0.789	0.731	0.853	0.855	1.000		
**ExtBehav**	0.799	0.707	0.965	0.979	0.929	1.000	
**AnxArous**	0.812	0.783	0.823	0.779	0.854	0.820	1.000
