# Supplementary material for: Preliminary validation of the Dutch version of the Posttraumatic stress disorder checklist for DSM-5 (PCL-5) after traumatic brain injury in a civilian population
Source: PLoS One. 2020 Apr 20;15(4):e0231857. doi: 10.1371/journal.pone.0231857 (PMC7170250; doi:10.1371/journal.pone.0231857)
Supplement: S4 Table — (PDF) [file pone.0231857.s004.pdf]

S4 Table. Correlations of depression and anxiety instruments with PCL-5

Author (year)	Language	Depression instrument	Correlation with PCL-5	Anxiety instrument	Correlation with PCL-5
Ashbaugh et al. (2016)[48]	English	CES-D	0.64	IES-R total<br>Subscale instrusion<br>Subscale avoidance<br>Subscale arousal	0.82<br>0.76<br>0.68<br>0.81
	French	CES-D	0.62	IES-R total<br>Subscale instrusion<br>Subscale avoidance<br>Subscale arousal	0.80<br>0.71<br>0.65<br>0.78
Boysan et al. (2017)[54]	Turkish	BDI	0.81	BAI	0.74
Hall et al. (2019)[56]	Filipino	PHQ-9	0.71	GAD-7	0.61
Ibrahim et al. (2018)[55]	Arab/Kurdish	DHSC	0.65		
Sveen et al. (2016)[50]	Swedish	MADRS	0.60		
