# Supplementary material for: Preliminary validation of the Dutch version of the Posttraumatic stress disorder checklist for DSM-5 (PCL-5) after traumatic brain injury in a civilian population
Source: PLoS One. 2020 Apr 20;15(4):e0231857. doi: 10.1371/journal.pone.0231857 (PMC7170250; doi:10.1371/journal.pone.0231857)
Supplement: S1 File — (PDF) [file pone.0231857.s005.pdf]

S5 File. Dutch translation of the PCL-5

Posttraumatic stress disorder checklist (PCL-5) – dutch version

Instructies: onderstaand vindt u een opsomming van problemen die mensen kunnen ondervinden na een stressvolle ervaring. Graag verzoeken wij u om ieder probleem zorgvuldig door te lezen en aansluitend te omcirkelen in hoeverre u last heeft gehad van deze problemen in de afgelopen maand.

Hoe vaak heeft u in de afgelopen maand last gehad van:	Helemaal niet	Af en toe	Gemiddeld	Vaak	Zeer vaak
1. Herhaalde, storende en ongewenste herinneringen aan de stressvolle ervaring?	0	1	2	3	4
2. Herhaalde, storende dromen over de stressvolle ervaring?	0	1	2	3	4
3. Plots gevoel of gedrag alsof de stressvolle ervaring zich opnieuw voordeed (alsof u weer in die situatie was en het opnieuw beleefde)?	0	1	2	3	4
4. Het gevoel erg overstuur te zijn wanneer u werd herinnerd aan de stressvolle ervaring?	0	1	2	3	4
5. Hevige lichamelijke reacties wanneer u werd herinnerd aan de stressvolle gebeurtenis (vb. hartkloppingen, moeite met ademhalen, zweten)?	0	1	2	3	4
6. Vermijden van herinneringen, gedachten of gevoelens die te maken ehbben met de stressvolle ervaring?	0	1	2	3	4
7. Vermijden van omgevingsfactoren die herinneren aan de stressvolle ervaring (vb. mensen, plaatsen, gesprekken, activiteiten, objecten of situaties)?	0	1	2	3	4
8. Moeite hebben met het herinneren van belangrijke zaken die te maken hebben met de stressvolle ervaring?	0	1	2	3	4
9. Hebben van sterke negatieve gedachten en overtuigingen over uzelf, anderen of de wereld (vb. ik ben een slecht mens, er is iets ernstig mis	0	1	2	3	4

met mij, ik kan niemand vertrouwen, de wereld is een gevaarlijke plek)?					
10. Uzelf of iemand anders de schuld geven voor de stressvolle ervaring of wat daarna gebeurde?	0	1	2	3	4
11. Hebben van sterke negatieve gevoelens zoals angst, afschuw, woede, schuld of schaamte?	0	1	2	3	4
12. Afgenomen interesse in activiteiten waar u voorheen van kon genieten?	0	1	2	3	4
13. Een gevoel van afstand t.o.v. andere mensen of buitengesloten zijn?	0	1	2	3	4
14. Moeite hebben met het voelen van positieve emoties (vb. blij zijn of liefde voelen voor vrienden en familie)?	0	1	2	3	4
15. Prikkelbaarheid, woede-uitbarstingen of agressief gedrag?	0	1	2	3	4
16. Risicovol gedrag vertonen of dingen doen die u schade zouden kunnen toebrengen?	0	1	2	3	4
17. "Superalert", extra oplettend of op uw hoede zijn?	0	1	2	3	4
18. Onrustig zijn of makkelijk schrikken?	0	1	2	3	4
19. Moeite hebben met concentreren?	0	1	2	3	4
20. Moeite hebben met in slaap vallen of doorslapen?	0	1	2	3	4

© F. Weathers et al. (8/14/2013)
