# Supplementary material for: Preliminary validation of the Dutch version of the Posttraumatic stress disorder checklist for DSM-5 (PCL-5) after traumatic brain injury in a civilian population
Source: PLoS One. 2020 Apr 20;15(4):e0231857. doi: 10.1371/journal.pone.0231857 (PMC7170250; doi:10.1371/journal.pone.0231857)
Supplement: S2 File — (PDF) [file pone.0231857.s006.pdf]

National Center for PTSD
POSTTRAUMATIC STRESS DISORDER

The PTSD Checklist for *DSM-5*

Version date: 14 August 2013

Reference: Weathers, F. W., Litz, B. T., Keane, T. M., Palmieri, P. A., Marx, B. P., & Schnurr, P. P. (2013). *The PTSD Checklist for DSM-5 (PCL-5) – Standard* [Measurement instrument]. Available from http://www.ptsd.va.gov/

URL: http://www.ptsd.va.gov/professional/assessment/adult-sr/ptsd-checklist.asp

This page intentionally left blank

PCL-5

Instructions: Below is a list of problems that people sometimes have in response to a very stressful experience. Please read each problem carefully and then circle one of the numbers to the right to indicate how much you have been bothered by that problem in the past month.

In the past month, how much were you bothered by:	Not at all	A little bit	Moderately	Quite a bit	Extremely
1. Repeated, disturbing, and unwanted memories of the stressful experience?	0	1	2	3	4
2. Repeated, disturbing dreams of the stressful experience?	0	1	2	3	4
3. Suddenly feeling or acting as if the stressful experience were actually happening again (as if you were actually back there reliving it)?	0	1	2	3	4
4. Feeling very upset when something reminded you of the stressful experience?	0	1	2	3	4
5. Having strong physical reactions when something reminded you of the stressful experience (for example, heart pounding, trouble breathing, sweating)?	0	1	2	3	4
6. Avoiding memories, thoughts, or feelings related to the stressful experience?	0	1	2	3	4
7. Avoiding external reminders of the stressful experience (for example, people, places, conversations, activities, objects, or situations)?	0	1	2	3	4
8. Trouble remembering important parts of the stressful experience?	0	1	2	3	4
9. Having strong negative beliefs about yourself, other people, or the world (for example, having thoughts such as: I am bad, there is something seriously wrong with me, no one can be trusted, the world is completely dangerous)?	0	1	2	3	4
10. Blaming yourself or someone else for the stressful experience or what happened after it?	0	1	2	3	4
11. Having strong negative feelings such as fear, horror, anger, guilt, or shame?	0	1	2	3	4
12. Loss of interest in activities that you used to enjoy?	0	1	2	3	4
13. Feeling distant or cut off from other people?	0	1	2	3	4
14. Trouble experiencing positive feelings (for example, being unable to feel happiness or have loving feelings for people close to you)?	0	1	2	3	4
15. Irritable behavior, angry outbursts, or acting aggressively?	0	1	2	3	4
16. Taking too many risks or doing things that could cause you harm?	0	1	2	3	4
17. Being "superalert" or watchful or on guard?	0	1	2	3	4
18. Feeling jumpy or easily startled?	0	1	2	3	4
19. Having difficulty concentrating?	0	1	2	3	4
20. Trouble falling or staying asleep?	0	1	2	3	4
